# Expression of *TXNRD1, HSPA4L* and *ATP1B1* Genes Associated with the Freezability of Boar Sperm

**DOI:** 10.3390/ijms23169320

**Published:** 2022-08-18

**Authors:** Anna Mańkowska, Przemysław Gilun, Łukasz Zasiadczyk, Przemysław Sobiech, Leyland Fraser

**Affiliations:** 1 Department of Animal Biochemistry and Biotechnology, Faculty of Animal Bioengineering, University of Warmia and Mazury in Olsztyn, 10-719 Olsztyn, Poland; 2 Department of Local Physiological Regulations, Institute of Animal Reproduction and Food Research of the Polish Academy of Sciences, Bydgoska 7, 10-243 Olsztyn, Poland; 3 Internal Disease Unit, Department of Clinical Sciences, Faculty of Veterinary Medicine, University of Warmia and Mazury in Olsztyn, 10-719 Olsztyn, Poland

**Keywords:** boar, semen, freezability, stress-related genes, proteins

## Abstract

Cryopreservation is associated with increased oxidative stress, which is responsible for sperm damage. We analyzed the effect of cryopreservation on mRNA and protein expression of thioredoxin reductase 1 (*TXNRD1*), heat shock protein family A (HSP 70) member 4 like (*HSPA4L*) and sodium/potassium-transporting ATPase subunit beta-1 (*ATP1B1*) genes in boar sperm with different freezability. Boars were classified as having good and poor semen freezability (GSF and PSF, respectively), according to the assessment of post-thaw sperm motility. Total RNA was isolated from fresh pre-freeze (PF) and frozen-thawed (FT) sperm from five boars of the GSF and PSF groups, respectively. Quantification of *TXNRD1*, *HSPA4L* and *ATP1B1* gene expression was performed by RT-qPCR analysis. Proteins extracted from sperm were subjected to Western blotting and SDS-PAGE analyses. Poor freezability ejaculates were characterized by significantly higher relative mRNA expression levels of *TXNRD1* and *HSPA4L* in FT sperm compared with the fresh PF sperm. Furthermore, the relative mRNA expression level of *ATP1B1* was significantly higher in the fresh PF sperm of the GSF group. Western blotting analysis revealed significantly higher relative expression of TXNRD1 protein in the fresh PF sperm of the GSF group, while HSPA4L protein expression was markedly increased in FT sperm of the PSF group. Electrophoretic and densitometric analyses revealed a higher number of proteins in the fresh PF and FT sperm of the PSF and GSF groups, respectively. The results of this study indicate that *ATP1B1* mRNA expression in the fresh PF sperm is a promising cryotolerance marker, while the variations of TXNRD1 and HSPA4L protein expression in the fresh PF or FT sperm provide useful information that may help to elucidate their biological significance in cryo-damage.

## 1. Introduction

Cryopreservation has been considered to be an efficient method of preserving the genetic materials of different animal species [1,2]. Besides individual variations, cryo-induced oxidative stress has been one of the major factors affecting the post-thaw (PT) quality of boar semen [2,3,4,5].

It has been suggested that sperm RNAs are transcriptionally inactive; however, studies have shown that sperm transport specific functional RNAs into the oocyte at the time of fertilization, and these RNAs could contribute to early embryonic development [6,7,8,9]. Studies have demonstrated that cryopreservation has varying effects on the profiles of sperm mRNA transcripts, which could be used as markers for sperm functions [8,10,11,12,13,14]. Expression of *PRM1* (Protamine 1) and *PRM2* (Protamine 2), suggested as potential fertility markers, is significantly reduced in frozen-thawed (FT) human sperm [10], and significant changes in the mRNA expression and protein levels of epigenetic-related genes have been detected in FT boar sperm [11,14]. Evidence has shown that transcriptome modifications in FT bull sperm are associated with reduced fertility [12], and an abundance of differentially expressed (DE) genes that are highly overexpressed in FT bull sperm with poor motility [13].

In this study we selected three stress-related genes, thioredoxin reductase 1 (*TXNRD1*), heat shock protein family A member 4 like (*HSPA4L*) and ATPase Na+/K+ transporting beta-1 polypeptide (*ATP1B1*), which are implicated in various sperm functions, such as spermatogenesis, motility, hyperactivation, capacitation and osmoregulation [15,16,17,18,19,20]. In boar sperm transcriptome these genes were not differentially expressed (DE) between the good and poor freezability ejaculates [21]. Besides boar sperm transcriptome, *TXNRD1*, *HSPA4L* and *ATP1B1* have been detected in human and ovine sperm transcriptomes [22,23]. The thioredoxin family is implicated in a wide range of cellular processes, including antioxidant defense, signaling of apoptosis, redox control of protein function and transcription factor activity [24,25]. Thioredoxin reductases are a family of selenium-containing pyridine nucleotide–disulfide oxidoreductases that play a key role in redox signaling events [18,24]. Two thioredoxin reductases, TXNRD1 and TXNRD2, have been shown to play a crucial role in maintaining thioredoxins in their reduced (active) state and are components of the cytosolic and mitochondrial thioredoxin systems, respectively [18]. It has been reported that reduced TXNRD1 content in asthenozoospermic sperm is associated with increased reactive oxygen species (ROS) generation, apoptosis and the number of immature sperm [26]. Moreover, HSPA4L is a member of the heat shock protein family (HSP110) that performs many functions, such as molecular chaperones that are synthesized under stress conditions to assist in protein folding, assembly and transport [15,16,27]. Evidence has shown that HSPA4L is required for normal spermatogenesis, and is produced under environmental stress and pathological or certain physiological conditions to protect cells from damage [15,16]. The HSPA4L protein is highly expressed in different spermatogenic cells and appears to play a role in osmotolerance, male fertility and sperm motility [15,23]. The protein encoded by the *ATP1B1* gene belongs to the family of Na+/K+–ATPase pumps, a mechanism responsible for maintaining the electrochemical gradient ions across the cellular membrane that could be disturbed by osmotic-induced stress [17,28,29]. The ATP1B1 protein has been detected in epididymal sperm of bulls and swamp buffalos [30,31], and its significantly high expression in bull epididymal sperm was associated with good semen freezability [30,31].

In this study, we hypothesized that the differential expression of the stress-related genes (*TXNRD1*, *HSPA4L* and *ATP1B1*) in boar sperm might be associated with cryotolerance. The main objective of the study was to determine the mRNA and protein expression levels of *TXNRD1*, *HSPA4L* and *ATP1B1* in fresh pre-freeze (PF) and FT sperm from boars classified as having good and poor semen freezability (GSF and PSF, respectively). In addition, the identification of the electrophoretic profiles of sperm proteins associated with freezability was performed in this study. 

## 2. Results

### 2.1. Semen Quality Assessment

The quality characteristics of the fresh PF semen did not differ (*p* > 0.05) between the GSF and PSF groups (Table 1). ANOVA results showed significant boar effects (*p* < 0.05) on the quality characteristics of FT sperm (Supplementary Table S1). We classified five boars each of the GSF (Boars no. 1–5) and PSF groups (Boars no. 6–10), according to post-thaw total motility (TMOT). Boars showing more than 30% (>30%) post-thaw TMOT were assigned to the GSF group, whereas those with TMOT less than 30% (<30%) were allocated to the PSF group (Table 2).

Besides post-thaw TMOT and progressive motility (PMOT), motion parameters analyzed by the computer-assisted sperm analysis (CASA) system showed higher (*p* < 0.05) PT values of velocity straight line (VSL), velocity average path (VAP) and velocity curvilinear (VCL), straightness (STR) and linearity (LIN) for the GSF group than the PSF group (Table 2). Likewise, the percentages of FT sperm with mitochondrial membrane potential (MMP), plasma membrane integrity (PMI), NAR acrosome integrity and DNA integrity were significantly higher (*p* < 0.05) in the GSF group than in the PSF group (Table 2). 

### 2.2. Analysis of Gene Expression

**Figure 1:**
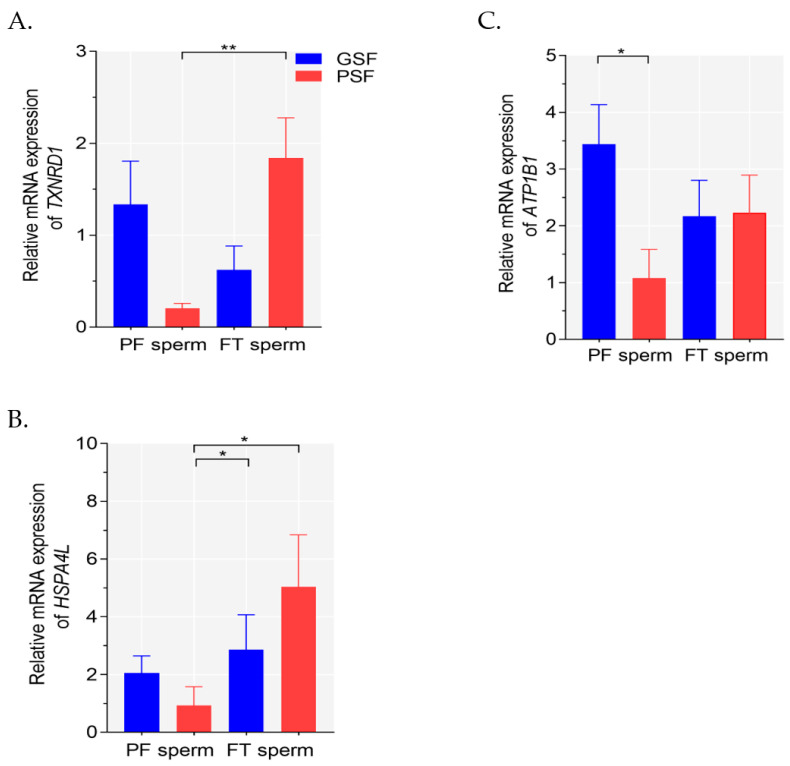
Relative mRNA expression of (**A**) *TXNRD1*, (**B**) *HSPA4L* and (**C**) *ATP1B1* in fresh pre-freeze (PF) and frozen-thawed (FT) sperm from boars of the good and poor semen freezability (GSF and PSF, respectively) groups. Values are presented as the mean ± SEM. Expression values of *TXNRD1*, *HSPA4L* and *ATP1B1* were normalized against the glyceraldehyde-3-phosphate dehydrogenase (GAPDH) value of the control. Significant at * *p* < 0.05, and ** *p* < 0.01 after the Bonferroni correction.

There were wide variations in the mRNA expression levels of *TXNRD1*, *HSPA4L* and *ATP1B1* in the fresh PF or FT sperm among the boars. It was found that there were significant effects on *TXNRD1* mRNA expression among the groups (Kruskal–Wallis ANOVA, *df* = 3, *p* < 0.011). FT sperm exhibited significantly higher (*p* < 0.05) *TXNRD1* mRNA expression compared with the fresh PF sperm of the PSF group (Figure 1A). In contrast, no significant differences (*p* > 0.05) were found between the fresh PF and FT sperm of the GSF group (Figure 1A). Significant variations in *HSPA4L* mRNA expression were observed among the groups (Kruskal–Wallis ANOVA, *df* = 3, *p* < 0.012). *HSPA4L* mRNA expression levels in FT sperm of the GSF and PSF groups were significantly higher (*p* < 0.05, respectively) than in the fresh PF sperm of the PSF group (Figure 1B). Significant treatment effects on *ATP1B1* mRNA expression levels were observed among the groups (Kruskal–Wallis ANOVA, *df* = 3, *p* < 0.047). Furthermore, the fresh PF sperm of GSF group exhibited approximately 3-fold higher (*p* < 0.05) *ATP1B1* mRNA expression compared with the PSF group (Figure 1C). 

### 2.3. Gene Ontology (GO) Enrichment Analysis and KEGG Pathways

The GO mitochondrial function (GO:MF) terms for *TXNRD1* and *ATP1B1* were represented by glutathione oxidoreductase activity (GO:0097573) and ATPase activator activity (GO:0001671) (Supplementary Table S2A). The GO biological process (GO:BP) term for *HSPA4L* was represented by novo centriole assembly (GO:0097742), while the GO:BP term for *TXNRD1* and *ATP1B1* was represented by cellular homeostasis (GO:0019725) (Supplementary Table S2B). Most of the GO cellular components GO:CC terms were represented by *ATP1B1*, such as ATPase-dependent transmembrane transport complex (GO:0098533) (Supplementary Table S2C). *TXNRD1*, *HSPA4L* and *ATP1B1* were assigned to different Kyoto Encyclopedia of Genes and Genomes (KEGG) pathways, such as selenocompound metabolism (KEGG:00450), FoxO signaling pathway (KEGG:04068) and thyroid hormone synthesis (KEGG:04918), respectively (Supplementary Table S2D).

### 2.4. Western Blotting Analysis

**Figure 2:**
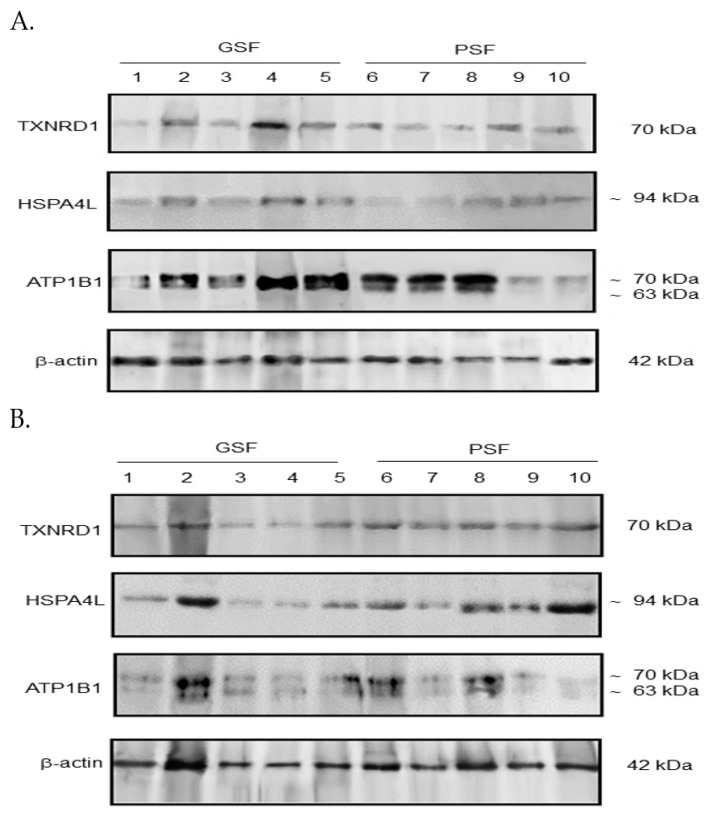
Western blotting analysis showing protein expression of TXNRD1, HSPA4L and ATP1B1 in (**A**) the fresh pre-freeze (PF) and (**B**) frozen-thawed (FT) sperm from boars of the good and poor semen freezability (GSF and PSF, respectively) groups.

Western blotting analysis showed significant variations in the expression of TXNRD1 (Kruskal–Wallis ANOVA, *df* = 3, *p* < 0.002), HSPA4L (Kruskal–Wallis ANOVA, *df* = 3, *p* < 0.008) and ATP1B1 (Kruskal–Wallis ANOVA, *df* = 3, *p* < 0.001) proteins among the groups. Blots showing TXNRD1, HSPA4L and ATP1B1 protein expression in the fresh PF and FT sperm are indicated in Figure 2A and Figure 2B, respectively. 

Bands representing 70 kDa for TXNRD1, and approximately 94 kDa (~94 kDa) for HSPA4L were detected in the lysates either from the fresh PF sperm (Figure 2A) or FT sperm (Figure 2B). We detected two protein bands (with molecular weights ~63 kDa and ~70 kDa) for ATP1B1 in the fresh PF and FT sperm (Figure 2A and Figure 2B, respectively). Analysis showed variations in the expression levels of the analyzed proteins in the PF and FT sperm among boars (Figure 2A and Figure 2B, respectively). 

**Figure 3:**
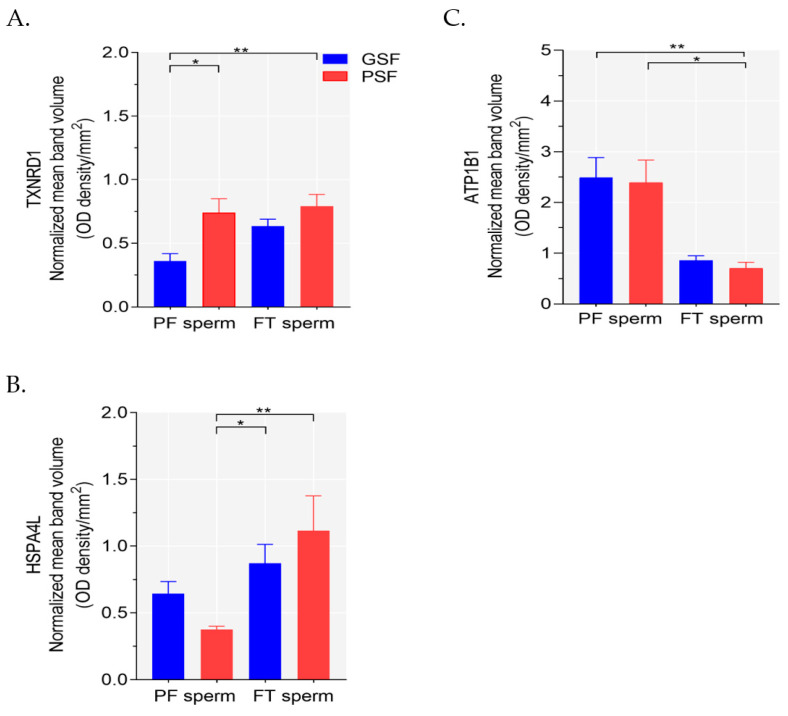
Relative expression of (**A**) TXNRD1, (**B**) HSPA4L and (**C**) ATP1B1 proteins in the fresh pre-freeze (PF) and frozen-thawed (FT) sperm from boars of the good and poor semen freezability (GSF and PSF, respectively) groups. Values represent the mean ± SEM. β-actin was used as the control to normalize the relative expression of the analyzed proteins. Significant at * *p* < 0.05, and ** *p* < 0.01 after the Bonferroni correction.

The fresh PF and FT sperm of the PSF group exhibited relatively higher (*p* < 0.05 and *p* < 0.01, respectively) TXNRD1 protein expression compared with the fresh PF of the GSF group (Figure 3A). It was found that the FT sperm of the GSF and PSF groups exhibited higher (*p* < 0.05 and *p* < 0.01, respectively) HSPA4L protein expression than the fresh PF sperm of the PSF group (Figure 3B). Furthermore, the fresh PF sperm of the GSF and PSF groups exhibited relatively higher (*p* < 0.01 and *p* < 0.05, respectively) expression of ATP1B1 protein (Figure 3C). 

### 2.5. Protein–Protein Interaction (PPI) Networks

**Figure 4:**
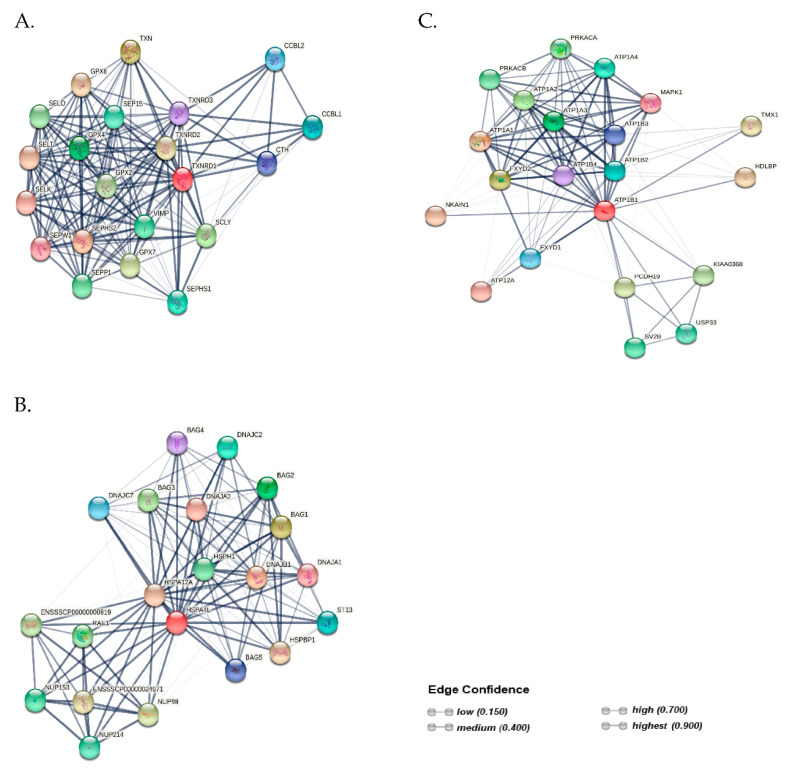
Protein–protein interaction (PPI) network visualized by STRING software. The nodes indicate proteins, and edges indicate the number of interactions for (**A**) TXNRD1, (**B**) HSPA4L and (**C**) ATP1B1.

The predicted PPI networks, generated by the STRING database v11.5, showed that TXNRD1, HSPA4L and ATP1B could interact and co-regulate the functions of different proteins involved in various biological processes (Figure 4A–C). Color saturation of the edges represented both functional and physical protein associations. A total of 21 nodes and 174 edges were identified for TXNRD1 (Figure 4A). Furthermore, a total of 21 nodes and 133 edges were constructed for HSPA4L, which comprised five BAG (Bcl-2 associated athanogene) cochaperones (Figure 4B). The network-based associations showed that the ATP1B1 protein was constructed with 21 nodes and 113 edges, and interacted with nine different proteins of the glycoprotein subunits of Na+/K+–ATPase (Figure 4C).

### 2.6. SDS-PAGE Analysis

**Figure 5:**
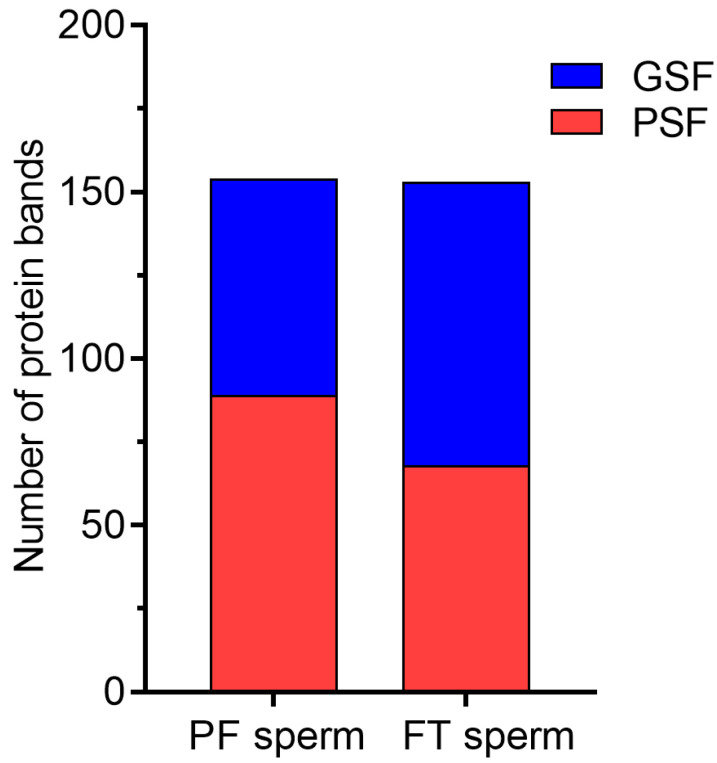
The average number of protein bands in the fresh pre-freeze (PF) and frozen-thawed (FT) sperm following sodium dodecyl sulfate-polyacrylamide gel electrophoresis (SDS-PAGE) and densitometry analyses.

In most of the boars, electrophoretic and densitometric analyses showed variations in the protein profiles between the fresh PF and FT sperm (Supplementary Figure S1). Protein abundance was more marked in the fresh PF sperm of the PSF group compared with the GSF group (89 vs. 65, respectively), while the FT sperm of the GSF group revealed a higher number of proteins (85 and 68, respectively), as shown in Figure 5. In general, cryopreservation resulted in the appearance of 40 and 16 additional proteins in the GSF (Supplementary Figure S1A) and PSF groups (Supplementary Figure S1B), respectively. The electrophoretic profiles of the FT sperm of three boars (Boars no. 1–3) showed the appearance of ten to eleven additional proteins, with molecular weights ranging from 14 to 137 kDa, while seven protein bands of 15–117 kDa were detected in the FT sperm of Boar no. 4 (Supplementary Figure S1A). Furthermore, the SDS-PAGE profiles of the FT sperm from one boar of the PSF group (Boar no. 9) showed the appearance of six additional protein bands of 17–103 kDa (Supplementary Figure S1B). 

**Figure 6:**
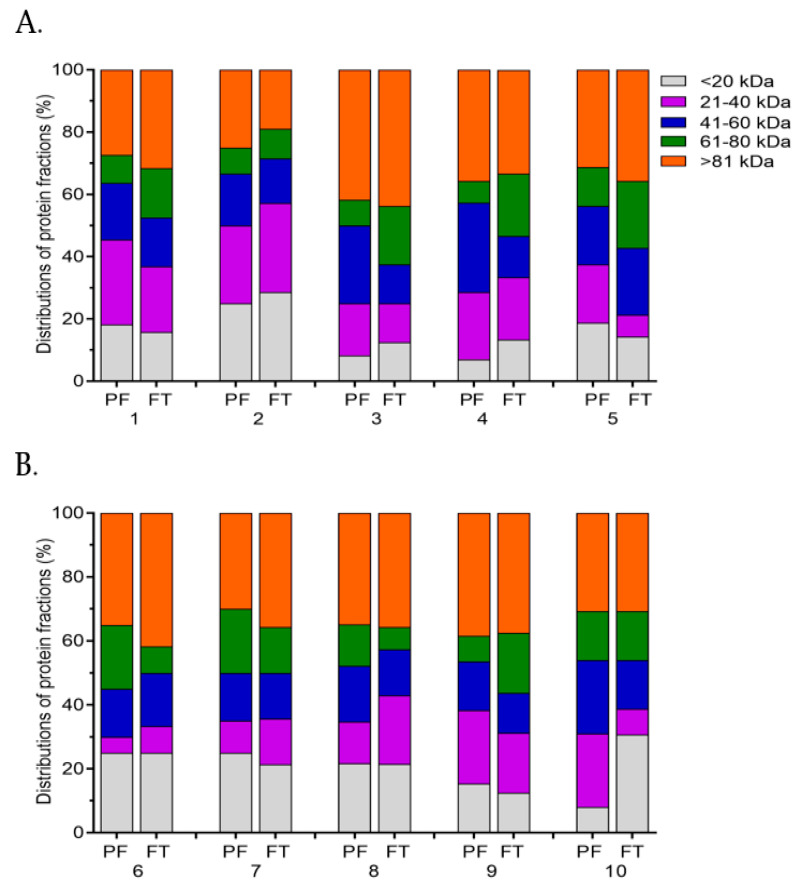
Distributions of the protein fractions in the fresh pre-freeze (PF) and frozen-thawed (FT) sperm from boars differing in freezability. (**A**) Protein fractions in the fresh PF and FT sperm from boars of the good semen freezability (GSF) group (Boars no. 1–5). (**B**) Protein fractions in the fresh PF and FT sperm from boars of the poor semen freezability (PSF) group (Boars no. 6–10).

In general, the SDS-PAGE and densitometric analyses showed variations in the distributions of the protein fractions among the boars (Figure 6A,B). Most of the boars of the GSF group showed a higher distribution (27.0–28.6%) of proteins of 21–40 kDa (21–40 kDa) in either the fresh PF or FT sperm (Figure 6A). Likewise, proteins with molecular weights lower than 20 kDa (<20 kDa) revealed a higher distribution (21.4–30.7%) in the fresh PF or FT sperm from three boars of the PSF group (Figure 6B). 

It was found that Boars nos. 3 and 4 showed approximately 2-fold higher distribution of proteins of 41–60 kDa (41–60 kDa) and 61–80 kDa (61–80 kDa) in the fresh PF and FT sperm, respectively (Figure 6A). Variations in the protein distribution groups of 41–60 kDa and 61–80 kDa among the boars of the PSF group were less marked between the fresh PF and FT sperm (Figure 6B). Also, the distribution of the proteins with molecular weight higher than 81 kDa (>81 kDa) was not markedly different between the fresh PF and FT sperm, regardless of the freezability group (Figure 6A,B).

## 3. Discussion

To the best of our knowledge, this study is the first to demonstrate that cryopreservation has varying effects on the relative mRNA expression levels of *TXNRD1*, *HSPA4L* and *ATP1B1* in boar sperm. Unexpectedly, while there were no marked differences in the mRNA expression of *TXNRD1* between the fresh PF and FT sperm of the GSF group, significantly higher expression of the gene was detected in FT sperm of the PSF group. Although the underlying mechanism responsible for high post-thaw mRNA *TXNRD1* expression in poor freezability ejaculates is not yet fully understood, we hypothesize that this phenomenon might reflect increased oxidative stress, which could reduce the sperm cryotolerance. Accordingly, *TXNRD1* is implicated in the maintenance of the redox state of oxidized proteins by scavenging ROS, thereby protecting sperm cells against oxidative damage [18]. However, the mechanisms responsible for the increased content of *TXNRD1* mRNA in FT sperm from poor freezability ejaculates remain unclear and would require further investigation.

Similar to mRNA *TXNRD1* expression, significantly higher mRNA expression of *HSPA4L* was detected in FT sperm of the PSF group. Evidence has shown that HSPA4L functions as a molecular chaperone and provides protection against oxidative stress in sperm [15,16]. Transcriptome analysis of FT bull sperm detected the up-regulation of four DE genes, such as ribosomal protein L31 (*RPLC*), glutamate-cysteine ligase catalytic subunit (*GCLC*), protein kinase C epsilon type (*PRKCE*) and proteolipid protein 1 (*PLP1*); however, the up-regulation of *GCLC* was suggested to be a protective response of the sperm to cold shock and oxidative stress incurred following freezing-thawing [12]. Moreover, transcriptome profiling of FT bull sperm revealed that transcripts regulating reactive oxygen species (ROS) production and maintenance of the physiological function of the cytoskeletal proteins were highly expressed in ejaculates with poor motility compared with those characterized by good motility [13]. Furthermore, the expression levels of genes encoding cold shock protein A (*CspA*), heat shock protein 60 (*HSP60*) and heat shock protein 10 (*HSP10*) were significantly increased in FT bull sperm [32]. Moreover, it has been demonstrated that cryopreservation induced alterations in the mRNA expression of epigenetic-related genes, resulting in the compromised functions of FT boar sperm [14]; however, it has been suggested that methylation modifications might not be the main factors responsible for increased mRNA expression of heat shock protein 70 (*HSP70*) and heat shock protein 90 (*HSP90*) in FT sperm [33]. Also, in the case of bull sperm transcriptome it was suggested that the abundant expression of transcript cytochrome oxidase subunit 7C (*COX7C*) in FT semen from low-fertility bulls might be due to the inefficient translation of the transcript [34]. In another study, transcriptome analysis of FT boar sperm showed that a marked increase in the expression of several genes that was associated with the signaling pathways, such as phosphoinositide-3-kinase–protein kinase B/Akt (PI3K-PKB/Akt) and cAMP signaling pathways [35]. Despite accumulating evidence, the mechanisms underlying the increase in mRNA expression levels of sperm-borne genes in FT semen from different animal species are unclear. In previous studies, it has been suggested that cryopreservation facilitated disturbance in the mRNA–protein interactions, thus rendering the mRNA molecules more susceptible to cryo-induced damage [10,33]. More importantly, the degradation of mRNA during the freezing-thawing procedure has been suggested as one of the factors that might be responsible for the reduced gene expression levels in FT sperm, because mature sperm probably lack active transcriptional machinery and do not have the ability to replace mRNA that is lost following cryopreservation [10,36]. More recently, we have confirmed that sperm from the poor freezability ejaculates are predisposed to increased oxidative stress [21]; however, it is unclear at this point whether higher *TXNRD1* or *HSPA4L* mRNA relative levels in FT sperm of the PSF group might be a protective response against cryo-induced oxidative stress. The mechanisms underlying the variations in mRNA expression levels of either gene between the fresh PF and FT sperm remain to be fully explored.

It is apparent that the higher mRNA expression of *HSPA4L* in FT sperm was accompanied by increased protein expression, regardless of the freezability group. The findings of the current study are not consistent with those of other studies, which have shown that low protein expression of HSPA4L was correlated with poor human sperm quality [15,16]. Interestingly, the response of sperm of both freezability groups to increased cryo-induced oxidative stress was accompanied by an abundance of HSPA4L protein, which did not contribute to an improvement in FT sperm quality, particularly in the PSF group. Similar observations have been reported in a recent study, which has shown that the abundance of HSP70 protein in FT water buffalo sperm was not accompanied by improved sperm functions, but rather by capacitation-like events, probably resulting in the synthesis of the chaperone protein [37]. Accumulating evidence has shown that oxidative stress, induced by ROS production following cryopreservation, compromises the sperm fertilizing ability [2,3,4,31]. It has been suggested that heat shock proteins are activated during stressful conditions in order to stimulate a pro-survival response of sperm cells during oxidative damage [16]. Accumulating evidence has shown that proteins that are involved in regulating stress-induced ROS production are more abundant in FT sperm [12,38,39]. Based on the findings of the current study, the abundance of the TXNRD1 and HSPA4L proteins in FT sperm did not provide protection against sperm cryo-damage, even though TXNRD1 and HSPA4L are closely associated with antioxidant protection (Figure 4A) and apoptotic rate (Figure 4B), respectively. The precise mechanism responsible for this phenomenon is currently unclear and warrants further investigation. 

ATP1B1 is an integral membrane protein that is involved in the regulation and maintenance of the electrochemical gradient of Na+ and K+ ions across the plasma membrane [28,29]. The functional Na+/K+–ATPase consists of an alpha subunit (110 kDa) and a beta subunit comprising 35–60 kDa, depending on glycosylation [17,28,29]. More importantly, ATP1B1 is one of the subunits of Na+/K+–ATPase that is regulated by the complex assembly of alpha/beta heterodimer (Figure 4C). Moreover, ATP1B1 is the subunit of ATP synthase that must undergo a conformational change to obtain energy, and its inactivation can affect various sperm functions, such as mitochondrial activity and motility [40]. Interestingly, the results of the current study show that high *ATP1B1* mRNA relative levels in the fresh PF sperm of the GSF group were not accompanied by an increase in sperm motility compared with the PSF group (Table 1). We suggest that the effect of mRNA *ATP1B1* expression in poor freezability ejaculates on the sperm functions is poorly understood. However, it is worth stressing that higher *ATP1B1* mRNA expression in the fresh PF sperm of the GSF group is consistent with the findings of our previous study, which confirmed that good freezability ejaculates exhibited high levels of NADH dehydrogenase subunit 6 (*ND6*) expression, a key component of the oxidative phosphorylation (OXPHOS) complex that is the most important pathway for ATP production [21]. We speculate that the reduced *ATP1B1* mRNA expression in the fresh PF semen of the PSF group could predispose sperm to reduced cryotolerance. Furthermore, we did not detect marked differences in the ATP1B1 protein levels in the fresh PF sperm between the freezability groups. The findings of this study are not consistent with those of a previous study, which demonstrated that increased post-thaw motility and mitochondrial function of epididymal sperm from the good freezability ejaculates were associated with higher expression of the ATP1B1 protein [30]. In the current study, we detected two protein bands of ATP1B1 (~63 kDa and ~70 kDa); however, we are not in a position to explain whether these isoforms could have resulted from glycosylation, even though a previous study reported the presence of glycosylated isoforms of ATP1B1 in the head membrane of bull sperm [17]. Furthermore, it appears that the significant loss of ATP1B1 protein in FT sperm of the PSF group indicates marked membrane damage, thus reiterating our findings that the poor freezability ejaculates are characterized by reduced cryo-damage. 

It is noteworthy that the mRNA expression of *TXNRD1* and *ATP1B1* was not concurrent with the protein abundance, particularly in FT sperm. However, evidence has indicated that, as a result of post-translational modifications in mature sperm, the abundance of proteins does not always reflect their relative expression levels of mRNA transcripts [7,41,42]. Presently, the mechanisms responsible for the variations in protein expression between the fresh PF and FT sperm have not been fully elucidated. However, several authors have suggested that such phenomena could be due to several factors, such as inefficient translation, post-translational modifications, enhanced phosphorylation or capacitation-like events [34,37,41,43,44]. According to Bogle et al. [45], variations in protein abundance between the fresh PF and FT sperm could be the consequence of a combination of protein degradation, post-translational processing, and alterations in the secondary or tertiary structures and/or translocation to other structures or outside the sperm cell. We do not know whether such phenomena could be a possible explanation for the abundance of TXNRD1 and HSPA4L proteins, as well as the electrophoretic protein fractions in FT sperm. Previous studies have demonstrated that the expression of several proteins that are involved in sperm capacitation, signal transduction, response to stress, energy status and sperm-oocyte fusion was increased in FT semen of boars, humans and rams [38,45,46]. In our previous study, we demonstrated that increased oxidative stress was triggered by the up-regulation of inflammatory and apoptosis-related genes in sperm from boars with low cryotolerance [21]. It should be emphasized that the abundance of TXNRD1 protein in the fresh PF sperm of the PSF group indicates that ejaculated sperm are predisposed to increased oxidative damage, which has been shown to be a characteristic feature of the poor freezability ejaculates [21]. However, this finding was not consistent with the expression levels of HSPA4L protein in the fresh PF sperm of the PSF group, suggesting that the chaperone protein might be responding to different stimuli [47]. In the current study, the lack of significant differences between the freezability groups regarding the post-thaw expression levels of TXNRD1, HSPA4l or ATP1B1 protein remains unclear, and suggests the need for further research to explain these findings. Even though our findings show marked differences in the electrophoretic protein profiles in the fresh PF sperm or PT sperm between the GSF and PSF groups, we did not analyze the association of the protein bands with sperm freezability. Moreover, a previous study has reported on the effects of proteins with different molecular weights on the cryotolerance of boar sperm [48]. According to Corcini et al. [48], proteins with molecular weights of 18, 19, 44, 65, 80 and 100 kDa could be potential markers for the sperm tolerance to the freezing-thawing procedure. Other studies have reported that specific sperm proteins were associated with good semen freezability and fertility [49,50]. It can be suggested that alterations in the protein profiles of FT sperm might lead directly to reduced fertility. We suggest that further studies will be needed to explain the significance of the protein fractions in the cryotolerance of boar sperm.

## 4. Materials and Methods

### 4.1. Chemicals and Media

Chemicals were bought from Sigma Chemical Company (St. Louis, MO, USA), unless otherwise stated. 

### 4.2. Animals and Semen Collections

Ten Polish large white (PLW) boars (with an average age of two years) were used in this study. 

The boars were stationed at the Cryopreservation laboratory, Faculty of Animal Bioengineering, University of Warmia and Mazury in Olsztyn. A total of five ejaculates were collected from each boar during the autumn–winter period, using the gloved-hand technique [21]. Water was available ad libitum. Semen samples with more than 70% sperm motility and 85% morphologically normal sperm were used in the study. All animal experiments were carried out in accordance with the guidelines set out by the Local Ethics Committee in Olsztyn (Poland). Approval of the Local Ethics Committee for experiments on boars (semen collection procedure) has not been required since 15 January 2015.

### 4.3. Semen Processing Procedure

Semen was frozen using lyophilized lipoprotein fractions of ostrich egg yolk (LPFo) according to a cryopreservation protocol [3,51,52]. Following the cooling of extended semen (2 h at 5 °C), samples were diluted (2:1) with a freezing extender (89.5 mL lactose-LPFo extender, 9 mL glycerol and 1.5 mL Orvus Es Paste), before being packaged in 10-mL sterilized aluminum tubes (500 × 10^6^ spermatozoa/mL). Samples were frozen in a programmable controlled-rate freezer (Ice Cube 1810, SY-LAB, Austria), using an appropriate cooling rate [51]. Prior to PT analysis, the samples were thawed in a water bath for 60 s at 50 °C, diluted in Beltsville Thawing Solution (BTS) and held in a water bath for 10 min at 37 °C.

### 4.4. Semen Quality Assessment

Assessment of quality characteristics was performed on the fresh PF and FT sperm.

#### 4.4.1. CASA Motility and Motion Parameters

Sperm motility and motion parameters were assessed with the CASA system (HTR-IVOS 12.3, Hamilton Thorne Biosciences, MA, USA) using the software settings described in a previous study [21]. The sperm parameters assessed by the CASA system included TMOT (%), PMOT (%), VSL (μm/s), VAP (μm/s), VCL (μm/s), STR (ratio of VSL/VAP × 100%), LIN (ratio of VSL/VCL × 100%), amplitude of lateral head displacement (ALH, μm) and beat cross frequency (BCF, Hz).

#### 4.4.2. Membrane Integrity Characteristics

The percentage of sperm with functional MMP was assessed with the fluorescent lipophilic cation JC-1 and propidium iodide (PI) fluorescent dyes [53]. Sperm samples were diluted (30 × 10^6^ spermatozoa/mL) in a HEPES-buffered solution, and incubated with JC-1 and PI solutions at 37 °C. A minimum of 100 cells per slide were examined at ×600 magnification under a fluorescence microscope (Olympus CH 30, Olympus Optical Co. Ltd. Tokyo, Japan), and sperm that exhibited orange–red fluorescence in the midpiece region were considered as viable sperm cells with functional mitochondria. Slides were analyzed in duplicate.

The percentage of sperm with PMI was assessed with the SYBR-14 and PI fluorescent probes, using the Live/Dead Sperm Viability Kit [54]. A minimum of 100 cells per slide were examined at ×600 magnification under a fluorescence microscope (Olympus CH 30), and two slides were evaluated per sample.

A modified Giemsa staining protocol was used to assess the percentage of sperm with NAR acrosome integrity [55,56]. Briefly, smears made on slides were fixed by immersion in formal saline solution before being stained with the Giemsa staining solution for 90 min at room temperature, prior to analysis. A minimum of 100 cells per slide, two slides per sample, were examined under a bright light microscope, equipped with oil-immersion lens at ×1000 magnification (Olympus BX 41, Olympus, Tokyo, Japan), and were classified as sperm with intact apical ridge or damaged apical ridge. 

#### 4.4.3. DNA Fragmentation

Sperm DNA fragmentation was assessed, using the comet assay [3,51]. Briefly, semen samples were washed by centrifugation (800× *g*, 5 min) and the pellets (10 × 10^6^ spermatozoa/mL) were re-suspended in a phosphate-buffered solution (PBS). Samples were placed on frosted microscope slides pre-coated with normal melting point agarose for lysing and RNase A treatment. Following treatment, samples were subjected to electrophoresis, before being fixed in 70% ethanol and stained with ethidium bromide. A minimum of 200 cells per slide were examined in random fields at 400× magnification under a fluorescence microscope (Olympus BX 41, Olympus, Tokyo, Japan), and sperm were classified as non-fragmented DNA (undamaged) and fragmented DNA (damaged) sperm cells. Slides were analyzed in duplicate.

### 4.5. Total RNA Isolation and Quantitative Real-Time PCR (RT-qPCR) Analysis

Total RNA was isolated from the fresh PF and FT sperm from ten boars, according to a previously described method [21]. At least three sperm samples (*n* = 3) from each boar were used for RNA isolation. Briefly, fresh PF and FT sperm samples (150 × 10^6^ spermatozoa/mL) were washed (5000× *g* for 5 min at 4 °C) in PBS, and sperm pellets were re-suspended in a hypotonic solution supplemented with 0.5% Triton X-100, prior to storage on ice for 20 min. Following this, samples were washed 2× in PBS, and were treated with a Lysis Buffer (PureLink RNA mini kit). The RNA extraction protocol was based on the utilization of the TRIzol/Pure Link™ RNA Mini kit (Invitrogen, Thermo Fisher Scientific Inc., Waltham, MA, USA), in conjunction with a Turbo DNase digestion procedure. The quantity, purity and 260/280 nm ratios of total RNA were examined by a Nanodrop Spectrophotometer (ND-1000, NanoDrop, Thermo Fisher Scientific Inc., Waltham, MA, USA), as described in a previous study [21].

The relative mRNA quantification of *TXNRD1*, *HSPA4L* and *ATP1B1* was performed using RT-qPCR analysis, according to a previously described method [57], with some modifications. The RNA samples were denatured for 10 min at 70 °C before the synthesis of cDNA. Two hundred nanograms of RNA were used as a template, and the reactions were performed using the High Fidelity cDNA Synthesis Kit (Roche Diagnostics International, Basel, Switzerland) with random hexamer, according to the manufacturer’s protocol. The cDNA synthesis was performed in a PCR Thermal Cycler (Labcycler, Sensoquest GmbH, Göttingen, Germany). All analyses were performed in duplicates. Aliquots of the RT products were diluted with nuclear-free water and used for RT-PCR analysis.

Amplifications were performed in a Real-Time PCR system (ABI 7900 HT, Applied Biosystems, Foster City, CA, USA) using the master mix volume that comprised 5 µL SYBR Green mix (Maxima SYBR Green/ROX qPCR Master Mix ×2, Thermo Fisher Scientific Inc., Waltham, MA, USA), 10 µM each of forward and reverse primers (2 µL) and 3 µL of template cDNA (equivalent amount of 3.75 ng mRNA). Amplifications were done in duplicates. Activation of DNA polymerase was achieved by incubating reactions for 10 min at 95 °C. The cDNA amplifications were performed by incubating the reactions for 40 cycles at 95 °C, for 15 s and 60 °C for 60 s. The Primer Express Software v3.0 (ABI 7900 HT, Applied Biosystems, Foster City, CA, USA) was used to design the primers used in the RT-PCR analysis. Primers used for the genes are shown in Table 3. Glyceraldehyde-3-phosphate dehydrogenase (GAPDH) was used as the reference gene [58]. The relative mRNA quantifications were performed by comparing the genes of interest with the reference gene (GAPDH), and are expressed as arbitrary units, using the Real Time PCR Miner algorithm [59].

### 4.6. GO Enrichment Analysis and KEGG Pathways

Enrichment analysis according to GO categories (MF, BF and CC) was performed, and the KEGG pathways of *TXNRD1*, *HSPA4L* and *ATP1B1* genes were analyzed, using the online tool g:Profiler (https://biit.cs.ut.ee/gprofiler/gost, accessed on 4 May 2022) [60]. The version of the g:Profiler was e105_eg52_p16_e84549f. Enrichment analysis was performed with the *sus scrofa* database, using the False Discovery Rate (FDR) of the Benjamini–Hochberg (BH) method for a significance threshold at 0.05.

### 4.7. Western Blotting Analysis

Sperm extracts were prepared from the fresh PF and FT sperm (50 × 10^6^ spermatozoa/mL), according to a previously described protocol [21,56]. Western blotting analysis was performed on sperm extracts from boars of the GSF group (Boars no. 1–5) and those of the PSF group (Boars no. 6–10) to quantify protein expression in TXNRD1, HSPA4L and ATP1B1. Protein samples (30 μg/lane) were separated by SDS-PAGE [61] and moved to Immobilon-P polyvinylidene fluoride (PVDF) membranes (Millipore, Bedford, MA, USA) for immunodetection. 

Electroblotting was performed according to a previously described method [62]. The procedure used for Western blotting analysis has been described in a previous study [21]. Following the blocking of the non-specific binding sites with 5% nonfat milk in Tris-buffered saline containing 0.05% *v*/*v* Tween 20 (TBST, MP Biomedicals LLC, Santa-Ana, CA, USA), blots were incubated overnight at 4 °C with the primary antibodies (Invitrogen, Thermo Fisher Scientific, Waltham, MA, USA) against rabbit polyclonal TXNRD1 (TrxR1) antibody (PA5-34685; 1:500), rabbit polyclonal HSPA4L antibody (PA5-44098; 1:500) and mouse monoclonal ATP1B1 antibody (MA3-930; 1:500). Rabbit beta-actin antibody (β-actin, A2066; 1:500; Sigma Chemical Company, St. Louis, MO, USA) was used as the loading control. After incubation, the membranes were incubated for 2 h at room temperature with anti-rabbit IgG, horseradish peroxidase (HRP)-linked secondary antibody (111-035-003; 1:1000; Jackson ImmunoResearch, Baltimore Pike, PA, USA). Protein detection was performed by enhanced chemiluminescence (ServaLight CL EOS Substrate kit (Serva, Heidelberg, Germany)). Images were captured with the G:BOX iChemi XR imaging system (SynGene, Cambridge, UK), and protein bands were quantified using MultiAnalyst 1.1 software (Bio-Rad Laboratories, Hercules, CA, USA). The molecular weights of the protein were determined using the Molecular weight standard (Pre-stained Protein Ladder™, Unstained Protein Standards, Bio-Rad Laboratories, Hercules, CA, USA). Values are expressed as the total signal intensity inside the boundary of a band measured in pixel intensity units/mm^2^—optical density (OD), using β-actin (Sigma Aldrich, Burlington, MA, USA) as the control to normalize the volume of the protein expression [63]. At least three replicates per boar were performed for the quantification of each analyzed gene in the fresh PF or FT sperm.

### 4.8. PPI Networks

We applied the Search Tool for the Retrieval of Interacting Genes database (STRING v11.5) available online (http://string-db.org, accessed on 12 August 2021) [64] to visualize the PPI networks for TXNRD1, HSPA4L and ATP1B1. 

### 4.9. Sodium Dodecyl Sulfate–Polyacrylamide Gel Electrophoresis (SDS-PAGE) Analysis

One dimensional SDS-PAGE and densitometry analyses were used to analyze the electrophoretic patterns of the proteins in the fresh PF and FT sperm, according to a previously described protocol [56], with some modifications. Briefly, protein samples (0.5 mg/mL) were precipitated on cold acetone (−20 °C), washed (14,000× *g* for 5 min), and the pellets were resuspended in a lysis buffer and heated for 5 min at 95 °C in a Thermo Block (TDB-120 thermostat (Biosan, Riga, Latvia). Electrophoresis was performed on 12% gels at 80 V for 15 min and then at 130 V for 1 h. Gels were stained with Coomassie Brilliant Blue R-250. The molecular masses of the protein bands were determined, PageRuler^TM^ Prestained Protein Ladder standards (Thermo Fisher Scientific Inc.), while the band intensity was assessed by densitometry analysis using MultiAnalyst 1.1 software (Bio-Rad Laboratories, Hercules, CA, USA). Analysis of the SDS-PAGE profiles included the total number of proteins and the difference in the electrophoretic profiles between the fresh PF sperm of the GSF and PSF groups or FT sperm of the GSF and PSF groups. Moreover, the protein fractions of the fresh PF sperm or FT sperm of each boar were categorized into five groups according to their molecular weights (<20 kDa, 21–40 kDa, 41–60 kDa, 61–80 kDa and >81 kDa) in order to assess the contribution of each fraction. 

### 4.10. Statistical Analysis

Statistical analysis was performed with the Statistica software package, version 13.3 (TIBCO Software Inc., Palo Alto, CA, USA; StatSoft Polska, Kraków, Poland) We used the ANOVA assumption (Shapiro–Wilk test) was used to check the normality of the data distribution of the sperm parameters followed by the application of the Levene’s test to examine for the homogeneity of variance. The general linear modeling (GLM) procedure was used for ANOVA analysis. A post-thaw TMOT cut-off threshold value of 30% was used for the classification of the freezability groups. Boars showing more than 30% (>30%) post-thaw TMOT were considered as having GSF, whereas those with TMOT lower than 30% (<30%) were classified as having PSF [21,65,66]. Assessment of the post-thaw semen quality showed that five boars (Boars no. 1–5) exhibited GSF, while five boars, (Boars no. 6–10) were considered having PSF. Descriptive variables of the sperm parameters are presented as the mean ± SEM, and the Student-t test was used for comparison between the GSF and PSF groups. The non-parametric Kruskal–Wallis ANOVA test was used to analyze VSL, VAP, VCL, ALH and BCF parameters, and the Mann–Whitney U test was used to compare the differences between the GSF and PSF groups. 

The IBM SPSS Statistics software package (IBM Corp. released 2020. IBM SPSS Statistics for Windows, version 27.0, IBM Corp. Armonk, NY, USA) was used to analyze protein and gene expression. The Kruskal–Wallis 1-way ANOVA with a multiple comparison test was used to analyze the protein and gene expression levels among the groups: fresh PF–GSF, fresh PF–PSF, FT–GSF and FT–PSF. The gene and protein expression levels are presented as the mean ± SEM, and significant values were adjusted by the Bonferroni correction for the multiple comparison test. Values were considered significant at *p* < 0.05.

## 5. Conclusions

Taken together, the results of the present study confirm that cryopreservation has varying effects on the mRNA expression levels of *TXNRD1*, *HSPA4L* and *ATP1B1* in boar sperm, particularly those from the poor freezability ejaculates. The findings of the present study confirm that the relative expression levels of *ATP1B1* mRNA in the fresh PF sperm could provide useful information to discriminate between the good and poor freezability ejaculates. We suggest that *ATP1B1* mRNA expression in the fresh PF sperm is a promising sperm cryotolerance marker, while the expression analysis of TXNRD1 and HSPA4L protein in FT sperm provides useful information that may help to elucidate their biological significance in cryo-damage. These findings support future investigations on the functional relevance of the mRNA and protein expression levels of TXNRD1, HSPA4L or ATP1B1 in the cryotolerance of boar sperm. 

## Figures and Tables

**Table 1:** Characteristics of fresh pre-freeze (PF) boar sperm prior to cryopreservation. Values are expressed as the mean ± SEM. GSF—good semen freezability; PSF—poor semen freezability.

Sperm Parameters	GSF (*n* = 25)	PSF (*n* = 25)	*p*-Value
Total motility (TMOT, %)	89.39 ± 0.69	87.38 ± 0.92	0.846
Progressive motility (PMOT, %)	63.84 ± 1.24	65.83 ± 1.06	0.231
Velocity straight line (VSL, μm/s)	75.90 ± 2.10	77.18 ± 2.01	0.584
Velocity average path (VAP, μm/s)	91.90 ± 1.76	93.58 ± 2.46	0.767
Velocity curvilinear (VCL, μm/s)	127.18 ± 4.20	125.18 ± 4.20	0.882
Straightness (STR, %)	81.35 ± 1.85	82.73 ± 1.29	0.535
Linearity (LIN, %)	60.32 ± 1.35	62.16 ± 1.45	0.355
Amplitude of lateral head displacement (ALH, μm)	7.16 ± 0.24	7.44 ± 0.19	0.657
Beat cross frequency (BCF, Hz)	30.40 ± 0.72	31.83 ± 0.95	0.231
Mitochondrial membrane potential (MMP, %)	87.51 ± 0.56	88.14 ± 0.67	0.664
Plasma membrane integrity (PMI, %)	88.17 ± 0.51	87.55 ± 1.05	0.721
NAR acrosome integrity (%)	91.88 ± 0.49	90.76 ± 0.71	0.185
DNA fragmentation (%)	2.23 ± 0.30	2.38 ± 0.26	0.698

Differences between the GSF and PSF groups for VSL, VAP, VCL, ALH and BCF parameters were compared, using the Mann–Whitney U test. Significant at *p* < 0.05; NAR—normal apical ridge.

**Table 2:** Post-thaw (PT) characteristics of boar sperm. Values are expressed as the mean ± SEM. GSF—good semen freezability; PSF—poor semen freezability.

Sperm Parameters	GSF (*n* = 25)	PSF (*n* = 25)	*p*-Value
Total motility (TMOT, %)	51.29 ± 1.47 ^a^	24.66 ± 0.82 ^b^	0.001
Progressive motility (PMOT, %)	40.71 ± 1.28 ^a^	17.17 ± 1.35 ^b^	0.001
Velocity straight line (VSL, μm/s)	60.97 ± 2.39 ^a^	46.15 ± 3.02 ^b^	0.001
Velocity average path (VAP, μm/s)	76.84 ± 2.56 ^a^	60.48 ± 3.16 ^b^	0.001
Velocity curvilinear (VCL, μm/s)	133.12 ± 3.50 ^a^	112.18 ± 2.79 ^b^	0.001
Straightness (STR, %)	79.10 ± 1.22 ^a^	75.20 ± 1.43 ^b^	0.042
Linearity (LIN, %)	46.07 ± 1.56 ^a^	40.75 ± 1.18 ^b^	0.049
Amplitude of lateral head displacement (ALH, μm)	5.49 ± 0.27 ^a^	6.42 ± 1.09 ^a^	0.375
Beat cross frequency (BCF, Hz)	21.70 ± 0.96 ^a^	28.23 ± 3.12 ^a^	0.072
Mitochondrial membrane potential (MMP, %)	52.10 ± 1.24 ^a^	31.61 ± 1.64 ^b^	0.001
Plasma membrane integrity (PMI, %)	51.24 ± 1.09 ^a^	36.46 ± 1.26 ^b^	0.001
NAR acrosome integrity (%)	51.45 ± 1.34 ^a^	38.50 ± 1.58 ^b^	0.001
DNA fragmentation (%)	7.05 ± 0.48 ^a^	12.90 ± 0.67 ^b^	0.001

Differences between the GSF and PSF groups for VSL, VAP, VCL, ALH and BCF parameters were compared, using the Mann–Whitney U test. Values with different letters (a and b) within the same row are significantly differed (*p* < 0.05); NAR—normal apical ridge.

**Table 3:** Primers used in reverse transcription real-time PCR analysis.

Gene	Ensemble Accession No.	Primer Sequence	Start (bp)	Stop (bp)	Amplicon Size (bp)
*TXNRD1*	NM_214154.3	F: 5′-CCAAACCCAAGGCGAAGTTT-3′ R: 5′-GTGTAAGCACGGGACACGC-3′	1589 1654	1608 1636	65
*HSPA4L*	XM_021101609.1	F: 5′-GGAGGTTGCGGCGCAG-3′ R: 5′-CTAGGGCAGTGCGTTGGG-3′	35 93	50 76	58
*ATP1B1*	NM_001001542.1	F: 5′-CCATCTTCAATCCCCGCA-3′ R: 5′-GCTTTTCCGCGGGCC-3′	89 143	106 129	54

bp—base pairs.

## Data Availability

The data presented in this study are available within the manuscript and in the Supplementary Materials. Further data can be provided upon request to the corresponding author.

## Supplementary Materials

The supporting information can be downloaded at: www.mdpi.com/article/10.3390/ijms23169320/s1.
