# Supplementary material for: Expression of TXNRD1, HSPA4L and ATP1B1 Genes Associated with the Freezability of Boar Sperm
Source: Int J Mol Sci. 2022 Aug 18;23(16):9320. doi: 10.3390/ijms23169320 (PMC9409117; doi:10.3390/ijms23169320)

Supplementary Figure S1. Sodium dodecyl sulfate-polyacrylamide gel electrophoresis (SDS-PAGE) profiles of proteins in fresh pre-freeze (PF) and frozen-thawed (FT) sperm from boars classified as having good and poor semen freezability (GSF and PSF, respectively). (A) GSF group (Boars no. 1 to 5). (B) PSF group (Boars no. 6 to 10). Samples were standardized (1.25 mg/ml), denatured, reduced and separated in 12% polyacrylamide gel. Arrow indicates the presence of an additional protein in either the fresh PF or FT sperm. MW – molecular weight standard

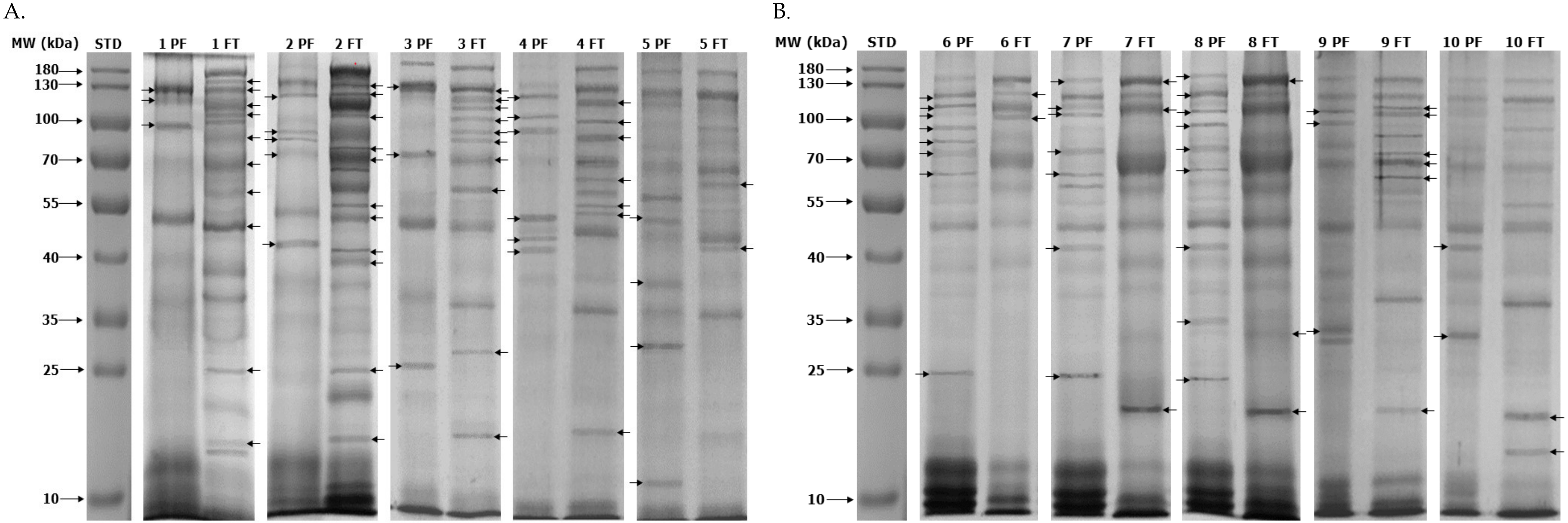

Supplement: Supplementary file 1 [file ijms-23-09320-s001.zip › ijms-1852801-supplementary/Supplementary Figure S1.pdf]
