# Supplementary material for: Expression of TXNRD1, HSPA4L and ATP1B1 Genes Associated with the Freezability of Boar Sperm
Source: Int J Mol Sci. 2022 Aug 18;23(16):9320. doi: 10.3390/ijms23169320 (PMC9409117; doi:10.3390/ijms23169320)
Supplement: Supplementary file 1 [file ijms-23-09320-s001.zip › ijms-1852801-supplementary/Supplementary Table S1.pdf]

Supplementary Table S1. ANOVA results showing boar effects on the quality characteristics of frozen-thawed (FT) sperm.

**Sperm parameters**	**ANOVA (df = 9)**	
	**F-value**	***p*–value**
Total motility (TMOT)	29.090	0.001
Progressive motility (PMOT)	18.784	0.001
Velocity straight line (VSL)	43.500	0.001
Velocity average path VAP)	40.086	0.001
Velocity curvilinear (VCL)	27.928	0.001
Straightness (STR)	7.842	0.001
Linearity (LIN)	14.184	0.001
Amplitude of lateral head displacement (ALH)	28.778	0.001
Beat cross frequency (BCF)	23.294	0.006
Mitochondrial membrane potential (MMP)	22.486	0.001
Plasma membrane integrity (PMI)	15.125	0.001
Normal apical ridge (NAR) acrosome integrity	8.929	0.001
DNA fragmentation	6.922	0.001

VSL, VAP, VCL, ALH and BCF parameters were analyzed with the Kruskal–Wallis ANOVA test. Significant at $p < 0.05$; df – degree of freedom
